# New insight into the role of the β3 subunit of the GABA_A_-R in development, behavior, body weight regulation, and anesthesia revealed by conditional gene knockout

**DOI:** 10.1186/1471-2202-8-85

**Published:** 2007-10-10

**Authors:** Carolyn Ferguson, Steven L Hardy, David F Werner, Stanley M Hileman, Timothy M DeLorey, Gregg E Homanics

**Affiliations:** 1 Department of Anesthesiology, University of Pittsburgh, Pittsburgh, PA 15261, USA; 2 Graduate Program in Molecular Pharmacology, University of Pittsburgh, Pittsburgh, PA 15261, USA; 3 Department of Physiology, West Virginia University, Morgantown, WV 26506, USA; 4 Molecular Research Institute, Palo Alto, CA 94303, USA; 5 Departments of Anesthesiology and Pharmacology, University of Pittsburgh, Pittsburgh, PA 15261, USA; 6 Department of Allied Health Diagnostics, Fairmont State University, Fairmont, WV 26554, USA

## Abstract

**Background:**

The β3 subunit of the γ-aminobutyric acid type A receptor (GABA_A_-R) has been reported to be important for palate formation, anesthetic action, and normal nervous system function. This subunit has also been implicated in the pathogenesis of Angelman syndrome and autism spectrum disorder. To further investigate involvement of this subunit, we previously produced mice with a global knockout of β3. However, developmental abnormalities, compensation, reduced viability, and numerous behavioral abnormalities limited the usefulness of that murine model. To overcome many of these limitations, a mouse line with a conditionally inactivated β3 gene was engineered.

**Results:**

Gene targeting and embryonic stem cell technologies were used to create mice in which exon 3 of the β3 subunit was flanked by loxP sites (i.e., floxed). Crossing the floxed β3 mice to a cre general deleter mouse line reproduced the phenotype of the previously described global knockout. Pan-neuronal knockout of β3 was achieved by crossing floxed β3 mice to Synapsin I-cre transgenic mice. Palate development was normal in pan-neuronal β3 knockouts but ~61% died as neonates. Survivors were overtly normal, fertile, and were less sensitive to etomidate. Forebrain selective knockout of β3 was achieved using α CamKII-cre transgenic mice. Palate development was normal in forebrain selective β3 knockout mice. These knockouts survived the neonatal period, but ~30% died between 15–25 days of age. Survivors had reduced reproductive fitness, reduced sensitivity to etomidate, were hyperactive, and some became obese.

**Conclusion:**

Conditional inactivation of the β3 gene revealed novel insight into the function of this GABA_A_-R subunit. The floxed β3 knockout mice described here will be very useful for conditional knockout studies to further investigate the role of the β3 subunit in development, ethanol and anesthetic action, normal physiology, and pathophysiologic processes.

## Background

γ-aminobutyric acid (GABA), acting via GABA type A receptors (GABA_A_-Rs), mediates the bulk of rapid inhibitory neurotransmission in the adult mammalian central nervous system. GABA_A_-Rs are pentameric chloride channels assembled from 19 different subunits, α 1–6, β1–3, γ1–3, δ, ε, π, ρ1–3, and θ[1]. Most native receptors are formed from 2α, 2β, and 1γ or 1δ subunits [2].

The β3 subunit is especially interesting because this subunit has been suggested to be a candidate gene for neurodevelopmental disorders such as Angelman Syndrome [3-5] and autism spectrum disorder [6-8]. β3 containing GABA_A_-R isoforms also are a crucial site of action for intravenous anesthetics [9-11] and ethanol [12], and an important receptor involved in developmental processes [13,14].

The β3 subunit is widely expressed in the developing rodent brain and spinal cord [15]. In the adult rodent, highest levels of β3 expression are restricted to hippocampus, cortex, olfactory bulb striatum, and lower levels are observed in numerous other brain regions [16,17].

To elucidate the role of the β3 subunit in physiology and pathophysiology, we previously created and characterized a mouse line that harbored a null allele of the β3 subunit [18]. These global knockout mice ubiquitously lacked the β3 protein throughout development in the whole animal. High levels of neonatal mortality [18] and compensatory adaptations were observed [19,20] that limited the utility of the model. To overcome the shortcomings of the global β3 knockout model, we have now successfully created a mutant β3 mouse line that can be used to create tissue-specific and/or developmentally regulated β3 knockouts. Here, the production and characterization of the conditional knockout model, and the use of this model to gain new insight into the role of β3 containing GABA_A_-Rs in development, behavior, body weight control, and anesthetic action is described.

## Results

### Gene targeting

**Figure 1:**
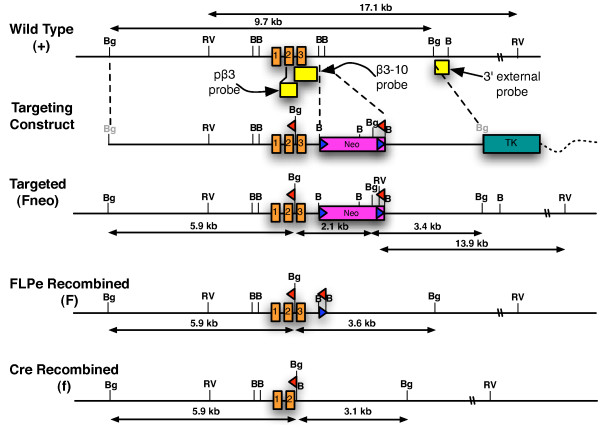
**Gene targeting strategy**. Diagram of the wildtype β3 locus illustrating the first three exons (orange boxes) and surrounding genomic DNA (thin, black line). Also shown are the DNA probes (yellow boxes) that were used for Southern blot analysis. The targeting construct included a neomycin (neo) cassette that was flanked by two frt sites (blue triangles), two loxP sites (red triangles), a thymidine kinase (TK) cassette, and plasmid vector backbone (broken, wavy line). During vector construction, the BglII restriction sites shown in grey lettering were destroyed. Also shown is a correctly targeted locus (Fneo), a locus following FLPe mediated deletion of the neo cassette (F), and a locus following Cre mediated deletion of exon 3 (f). Abbreviations: BamHI, B; BglII, Bg; EcoRV, RV.

A gene targeting construct (see Figure 1) was introduced into mouse embryonic stem cells to modify the β3 locus. A total of 91 embryonic stem cell clones were screened by Southern blot analysis to identify correctly targeted clones. Eight clones were correctly targeted. Gene targeting at the β3 locus created an EcoRV restriction fragment length polymorphism; a 3' probe that was external to the targeting construct hybridized to a 17.1 kb fragment from the wild type allele (designated β3^+^) and to a 13.9 kb fragment from a correctly targeted allele (designated as β3^Fneo^; see Figure 1). Retention of the 5' loxP site was verified by the presence of a 2.1 kb BglII restriction fragment that hybridized to the β3–10 probe. Correctly targeted clones were analyzed with several additional enzymes and probes and all results were consistent with targeting at the β3 locus (data not shown).

Of 4 correctly targeted clones injected into mouse blastocysts, chimeric mice from 2 clones transmitted the targeted β3 allele to the F1 generation following mating to C57BL/6J females. The results presented here were derived from clone 77S8.

### The unrecombined floxed β3 gene functioned normally

Heterozygous (β3^+/Fneo^) F1 mice were mated to an actin-FLPe transgenic deleter mouse line [21] to remove the neo gene by FLPe-mediated site-specific recombination. This produced mice that were heterozygous for the wild type and the floxed (flanked by loxP sites) β3 alleles and lacked the neo gene (β3^+/F^). These mice were interbred to produce wild type (β3^+/+^), heterozygous (β3^+/F^) and homozygous floxed (β3^F/F^) mice. β3^F/F ^mice were born at the expected frequency, were viable, did not have cleft palate, and were overtly indistinguishable from control littermates. This is in direct contrast to mice with global disruption of the β3 gene [3,14,18]; most of these mice died as neonates, had cleft palate, and those that survived had multiple behavioral abnormalities. Thus, the floxed β3 gene did not grossly have an adverse effect on function of the β3 gene.

### The recombined β3 gene is a null allele

β3^+/F ^mice were subsequently crossed to an actin-cre transgenic deleter mouse line [22] to recombine the floxed β3 allele and delete exon 3. Deletion of this 68 basepair exon was predicted to create a frameshift mutation and introduce a premature stop codon. The predicted product of the mutant β3 allele contains amino acids 1–58 of β3 followed by a novel 13 amino acid sequence. This product, which is devoid of the amino terminal 415 amino acids (β3 protein is normally 473 amino acids in length), should produce a nonfunctional product as all four of the putative transmembrane domains would be absent. Mice that were heterozygous for the cre recombined allele (β3^+/F^) were interbred to produce β3^+/+^, β3^+/F^, and homozygous recombined (β3^F/F^) mice. While the frequency of β3^F/F ^offspring was close to that expected by Mendelian genetics [actual 11 of 61 (18%) vs expected frequency of 25%], 10 of the 11 offspring died 2 days postnatally and the survivor at day 21. Fifty-four percent (6 of 11) of these mice had cleft palate. These results were consistent with the phenotype of global β3 knockouts that were produced by traditional gene targeting [18]. Thus, the recombined β3 allele functioned as a null allele following cre-mediated recombination.

### Production and analysis of pan-neuronal β3 knockout mice

Floxed β3 mice were also crossed to a synapsin I-cre (Syn-cre) transgenic mouse line [23,24] to produce neuron-specific conditional knockout mice. This cre expressing mouse line induces widespread recombination exclusively in neurons starting at embryonic day 12.5.

To investigate the effects of embryonic, pan-neuronal knockout of β3, pups were collected before birth (embryonic day 17.5–20.5) from β3^F/F^, cre- by β3^F/F^, cre+ mating pairs for genotype analysis. This study revealed that β3^F/F^, cre+ pups were present at a frequency that was similar to β3^F/F^, cre- pups (28/53 and 25/53, respectively). Visual analysis of the palates of a subset of these mice revealed that 0 of 9 β3^F/F^, cre+ pups examined in detail had cleft palate. Thus, we conclude that pan-neuronal knockout of β3 is compatible with *in utero *survival and normal palate development.

To investigate the effects of pan-neuronal knockout of β3 on postnatal survival and development, a genotypic analysis of weanling mice derived from β3^F/F^, cre- by β3^F/F^, cre+ mating pairs was performed. This study revealed that only ~28% (108/384) of the mice genotyped were cre+, in contrast to the ~72% (276/384) that were cre-. This differs from the expected 1:1 ratio. These results suggest that ~61% of the β3^F/F^, cre+ mice died before weaning. Many of the cre+ pups died within a few days of birth. Thus, we conclude that pan-neuronal inactivation of β3 by Syn-cre appears to result in a variably penetrant, non-cleft palate, neonatal lethality.

Gross behavior of the β3^F/F^, cre+ mice that survived beyond weaning were observed while in their home cage and during handling. The cre+ mice were overtly indistinguishable from control, cre- littermates. Unlike the global β3 knockouts [3,18], these conditional β3 knockouts did not display foot clasping behavior, hyperactivity, seizures, or tremors. In addition, both sexes of β3^F/F^, cre+ mice were found to be fertile and females displayed normal maternal care.

**Figure 2:**
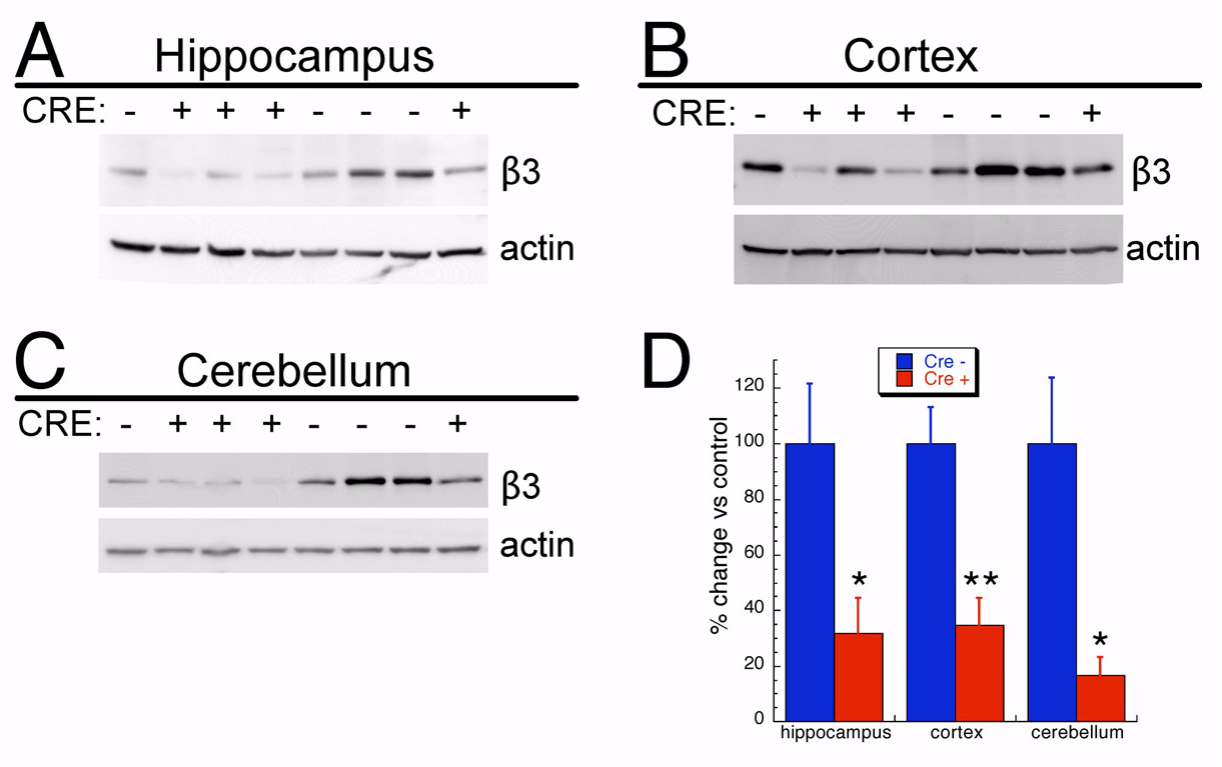
**Western blot analysis of pan-neuronal β3 knockouts**. Western blot analysis of the β3 subunit of the GABA_A_-R from individual 7–9 week old mice revealed dramatically reduced amounts of β3 in hippocampus (A), cortex (B) and cerebellum (C) of Syn-cre positive samples compared to cre negative control samples. All blots were reprobed for β-actin as a loading control. Shown are representative western blots. Five mice of each genotype were analyzed and all samples were analyzed on at least two different blots. D. Summary graph of western blot analysis demonstrating a significant (*, p < 0.05;**, p < 0.01) reduction in β3 protein in all brain regions examined. Data are expressed as mean ± SEM of percent change in band intensity relative to cre negative controls following normalization to actin.

To examine the degree to which the β3 gene product was reduced in various brain regions of the knockouts, western blot analysis of hippocampus, cortex, and cerebellum was performed using a β3 specific antibody. As shown in Figure 2, Syn-cre mediated knockout of β3 resulted in a dramatic reduction of β3 protein in all brain regions examined.

**Figure 3:**
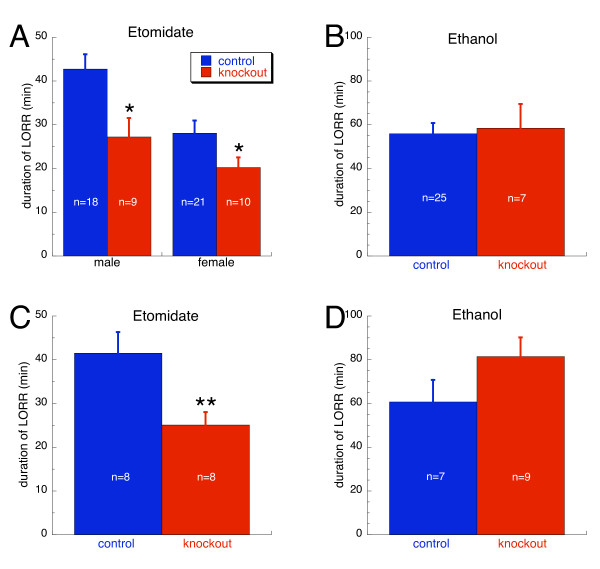
**Loss of righting reflex (LORR) assay**. A. Etomidate-induced LORR in pan-neuronal knockout mice was dependent on gender (p < 0.01). Therefore, male and female mice were analyzed separately. Both male and female pan-neuronal β3 knockouts were less sensitive to the sedative/hypnotic effects of etomidate (20 mg/kg) as evidenced by the reduced duration of the LORR compared to same sex controls. B. Ethanol-induced (3.5 g/kg) LORR did not differ between controls and pan-neuronal β3 knockouts. C. Etomidate-induced (20 mg/kg) LORR in forebrain selective β3 knockouts was not dependent on gender but knockouts were less sensitive compared to controls. D. Ethanol-induced (3.5 g/kg, i.p.) LORR of forebrain selective knockout mice did not differ compared to controls. *, p < 0.05; **, p ≤ 0.01.

Pan-neuronal knockout mice were tested for sensitivity to the intravenous anesthetic etomidate and ethanol using the standard loss of the righting reflex (LORR) assay. For etomidate, the analysis revealed a significant effect of gender (p < 0.01). Therefore, males and females were analyzed separately. This gender difference was also seen when etomidate was administered intravenously (data not shown). As shown in Figure 3A, both male and female knockouts were less sensitive to the sedative/hypnotic effects of etomidate as evidenced by the reduced duration of the LORR as compared to controls (p < 0.05). There was no effect of gender on ethanol-induced sleep time. Therefore, data were collapsed and analyzed together. Ethanol-induced sleep time did not differ between genotypes (Figure 3B).

### Production and analysis of forebrain selective β3 knockout mice

To produce a β3 knockout that is restricted only to postnatal forebrain neurons, the floxed β3 mice were crossed with an α CamKII-cre mouse line [25]. This mouse line has been reported to restrict recombination specifically to pyramidal neurons of the CA1 region of the hippocampus starting ~2 weeks after birth [25]. In a complementary report, a slightly broader pattern of recombination, deemed as "forebrain selective", that included the amygdala and cortex in addition to the hippocampus was described [26].

Mating of β3^F/F^, cre- by β3^F/F^, cre+ produced normal sized litters with no apparent neonatal mortality. However, some cre+ mice died between 15 and 25 days after birth, a time frame that coincides with expression of cre recombinase from the α CamKII-cre mouse line [25]. General observation of these mice discovered no abnormal phenotype, but interestingly, of 39 cre+ mice that died prematurely, 72% were male. At 4 weeks old, genotype analysis of surviving offspring revealed a ~30% reduction in cre+ mice (121/294) compared to cre- (173/294). Some of the cre+ mice that survived beyond weaning exhibited abnormal neurological behavior. These mice tended to be hyperresponsive to human contact, and at times appeared to be 'frozen' in their home cage, possibly having absence seizure-like behavior. This phenotype appeared to worsen with age and is similar to that observed in global β3 knockout mice [3].

**Figure 4:**
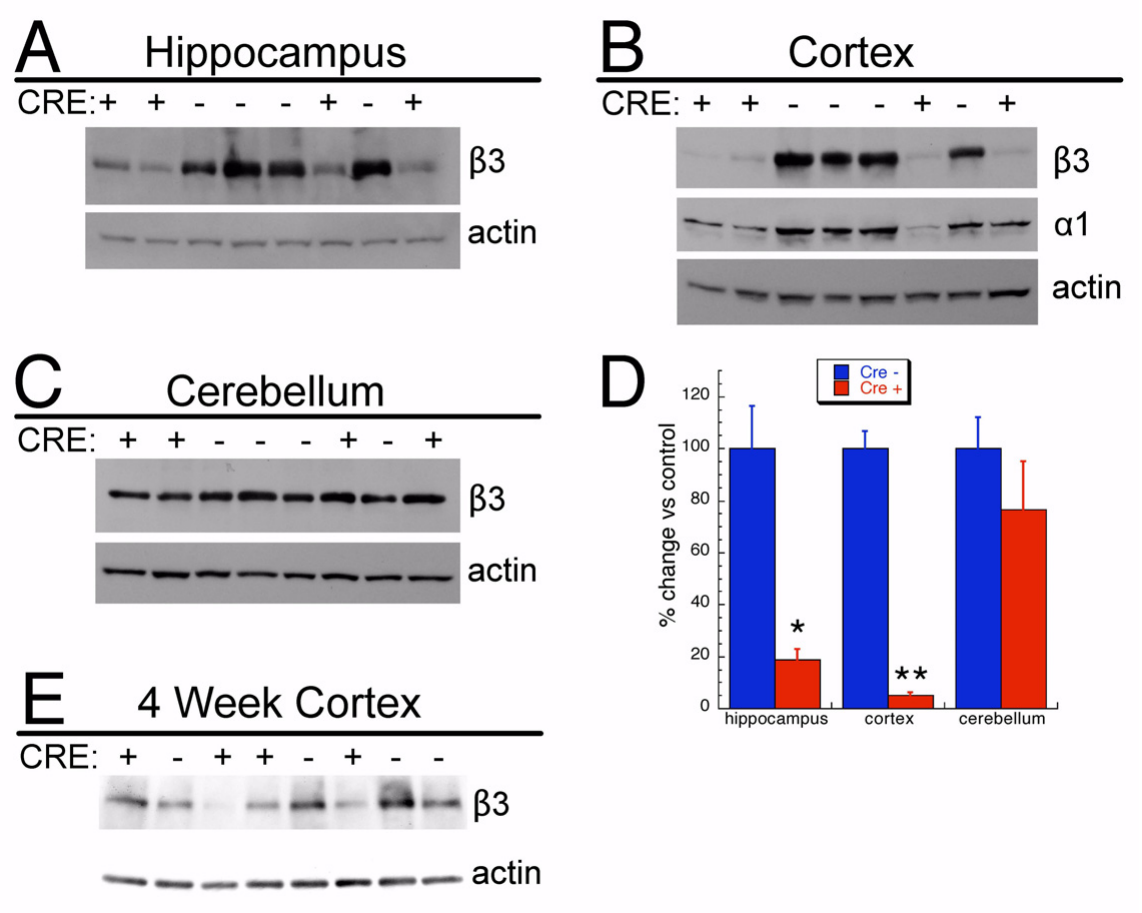
**Western blot analysis of forebrain selective neuronal β3 knockouts**. Western blot analysis of the β3 subunit of the GABA_A_-R from individual 13–16 week old mice revealed dramatically reduced amounts of β3 in hippocampus (A) and cortex (B) of α CamKII-cre positive samples compared to cre negative control samples. The amount of β3 in cerebellum (C) did not differ between genotypes. Analysis of cortex for the GABA_A_-R α1 subunit revealed a ~95% reduction in knockout samples compared to cre negative controls. All blots were reprobed for β-actin as a loading control. Shown are representative western blots. Four mice of each genotype were analyzed and all samples were analyzed on at least two different blots. D. Summary graph of western blot analysis demonstrating a significant (*, p ≤ 0.01; **, p < 0.001) reduction in β3 protein in hippocampus and cortex, but not in cerebellum. Data are expressed as mean ± SEM of percent change in band intensity relative to cre negative controls following normalization to actin. (E) Western blot analysis of β3 in cortex from individual 4 week old mice revealed dramatically reduced amounts of β3 in cre positive samples compared to cre negative control samples.

To examine the impact of α CamKII-cre mediated recombination on the β3 gene product, western blot analysis was conducted on hippocampus, cortex, cerebellum, and hypothalamus using a β3 specific antibody. As shown in Figure 4, β3 protein was dramatically reduced in hippocampus and was nearly completely ablated in cortex of cre+ mice compared to cre- controls. In contrast, the amount of β3 in cerebellum was not significantly reduced by the α CamKII-cre transgene. Cortex from 4 week old mice was also examined for the amount of β3. This analysis revealed a ~50% reduction in β3 in forebrain selective knockout mice compared to controls (Figure 4E).

Reproductive fitness of β3^F/F^, cre+ mice was tested by crossing to wild type mates. Both males and females were reproductively impaired. Two of six females that were test-mated consistently produced and cared for normal sized litters. The four other females either did not produce any offspring or produced litters infrequently and usually failed to care for pups. Male β3^F/F^, cre+ mice were test mated to superovulated females. None of the four cre+ mice produced copulation plugs, in contrast to 12 of 12 cre- mice that were tested for comparison. In addition, these four cre+ mice were housed with β3^F/+^, cre- females for 2–4.5 months. One male sired a seemingly normal number of litters during that time (n = 4), whereas the other three males each only sired a single litter. Thus, male reproductive fitness also seemed to be compromised.

**Figure 5:**
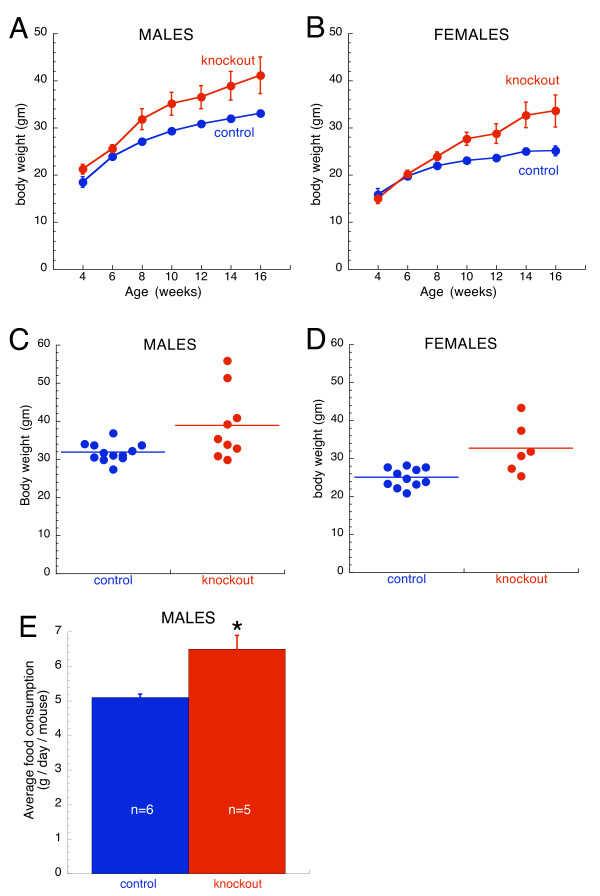
**Body weight and food consumption analyses of forebrain selective β3 knockouts**. Growth curves of (A) male and (B) female mice (n = 4–12 mice of each gender per genotype at each age). Body weight of knockout mice was greater than controls (repeated measures ANOVA: p < 0.01 for males; p < 0.05 for females). Plotted are means ± SEM. For data points without error bars, the bars are obscured by the symbol. Body weight of individual (C) male and (D) female mice at 14 weeks of age. The horizontal line indicates the group mean. Male and female knockouts were heavier than controls (p ≤ 0.05). Note the great variability in the body weight of knockout mice, including several obviously obese male animals that are ~1.75× heavier than controls. (E) Obese forebrain selective male knockout mice consumed more food than controls (*, p < 0.05). Shown is the average daily consumption per mouse ± SEM.

During the course of these studies, we also noticed that some, but not all, β3^F/F^, cre+ became obese in adulthood. To quantify this, the body weights of mice from 4–16 weeks of age were measured. As shown in Figure 5A, B, cre+ mice attained significantly greater body weights by ~8–10 weeks of age compared to cre- controls. Furthermore, analysis of the distribution of body weights revealed that there was considerable variability in the body weights of the cre+ mice. For example, at 14 weeks of age, while some cre+ animals had body weights that overlaped with controls, others had body weights that were 1.75× average control values (Figure 5C, D).

To find out if the obese mice also had a greater food intake, food consumption between knockout males and controls was compared. By 20 weeks of age, all surviving cre+ males were obese (47.7 ± 2.0, g ± SEMfor KO's vs. 33.9 ± 1.8 for controls). Average food consumption assessed over the course of 8 days was greater in knockouts compared to controls (p < 0.05; Figure 5E).

The forebrain selective knockout mice were also tested for sensitivity to etomidate and ethanol. No effect of gender was observed in either assay. As shown in Figure 3C, knockouts were less sensitive to the sedative/hypnotic effects of etomidate compared to controls (p ≤ 0.01). In contrast, ethanol-induced sleep time did not differ between genotypes (Figure 3D).

**Figure 6:**
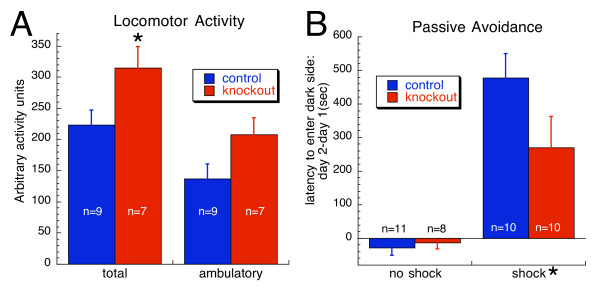
**Behavioral responses of forebrain selective β3 knockouts**. A. Spontaneous locomotor activity was measured and expressed in arbitrary units over a 10 min assessment period. Forebrain selective knockout mice were found to exhibit a statistically significant increase in total activity compared to cre- control mice. When the ambulatory component of total activity was examined, there was a trend for increased activity (p = 0.067). B. Passive avoidance. Shown is the latency to enter the dark side of the test apparatus expressed as the difference in latency between day 2 and day 1. Mice were exposed to a mild footshock (0.4 mA, 3 sec) on day 1. Separate groups of animals were also tested under identical conditions except they did not receive the footshock on day 1. Footshock increased the latency to enter (p ≤ 0.0001), but genotype and the interaction of genotype with footshock were not significant. *p < 0.05

Spontaneous locomotor activity testing revealed that forebrain selective knockout mice were more active than the Cre- control mice as evidenced by the greater total activity of knockouts (p < 0.05; Figure 6A). When only the ambulatory component of the total activity measurement was isolated for separate comparison, there was a trend for knockouts to exhibit greater activity (p = 0.067). During locomotor assessments mice were also closely observed for any indication of convulsive or absence-like behavior. No convulsive like behaviors were observed during testing, however one mouse was observed to have a convulsive seizure on a later date.

Mice were tested for learning and memory using a passive avoidance assay. As shown in Figure 6B, time to re-enter the dark side of the test apparatus was significantly increased by an aversive footshock (p < 0.0001). However, there was no effect of genotype or interaction of genotype with shock.

## Discussion

In this study a novel genetically engineered mouse line that was used to create GABA_A_-R β3 subunit conditional gene knockout mice was developed. This mouse line allowed further assessment of the involvement of the β3 GABA_A_-R subunit in formation of the palate, viability, body weight regulation, behavioral responses, and sedative/hypnotic drug sensitivity. Results are summarized and compared to global β3 knockouts in Table 1.

**Table 1:** Comparison of phenotypic abnormalities observed in gene targeted β3 gene knockout mice

***Phenotype***	***Global knockout***	Pan-neuronal knockout*	Forebrain selective knockout*
Time course of gene inactivation	Embryonic day 0 [18]	~Embryonic day 12.5+	~2 weeks postnatal
Tissue specificity	All cells [18]	Most neurons	Primarily forebrain neurons
Viability	~90% die as neonates [18]	~61% die as neonates	~30% die at 15–25 days of age
Palate	~55% with cleft [18]*	Normal	Normal
Overt behavior	Hyperactive; hyperresponsive [3, 18]	Normal	Moderate hyperactivity; jumpy
Locomotor activity	Increased [3]	Not tested	Increased
Motor coordination	Impaired [3]	Not tested	Not tested
Seizure-like activity	Multiple seizure types [3]	None observed	Occasional absence-like and convulsive seizures
EEG	Abnormal [3, 18]	Not tested	Not tested
Foot clasping	Present [18]	Absent	Absent
Fertility	Normal [18]	Normal	Reduced
Maternal behavior	Impaired [18]	Normal	Impaired
Body size	Runted until weaning but most attain normal size by adulthood [18]	Normal	Some become obese
Food intake	Not tested	Not tested	Increased
Etomidate LORR	Reduced [33]	Reduced	Reduced
Ethanol LORR	Normal [33]	Normal	Normal
Learning and memory	Impaired [3]	Not tested	Normal
Rest-activity cycles	Disturbed [3]	Not tested	Not tested

*, results from present study.

Cre recombinase mediated global knockout of β3 resulted in a high frequency (~55%) of cleft palate. This result agrees with studies that have reported a high frequency of cleft palate in mice that lack β3 due to traditional knockout of β3 [18] or radiation-induced deletion of β3 [27]. Furthermore, it has been demonstrated that ubiquitous expression of β3 alone on a β3 deficient background is sufficient to rescue this phenotype [14].

While these global knockout/transgenic studies clearly define a role for β3 in normal palate formation, they do not distinguish between a neuronal and nonneuronal site of action. To dissect the contribution of neuronal vs nonneuronal β3 in palate formation, a conditional knockout mouse line which lacked β3 in most neurons was developed. In these pan-neuronal knockout mice, cre-mediated recombination was under the control of the rat synapsin I promoter. Although the time course of cre-mediated inactivation of β3 in these mice was not examined in detail, this same Syn-cre transgenic mouse line has been demonstrated to induce recombination primarily in neurons of the central nervous system starting at approximately embryonic day 12.5 [23,24]. Cleft palate was not observed in this mouse line. These results lead us to suggest that neuronal β3 is not required for normal palate formation. This is consistent with the conclusions derived from a study where β3 was expressed only in neurons on a β3 knockout background [28]. That study demonstrated that neuronal expression of β3 was unable to rescue the animals from the clefting defect. Together, these results lead us to suggest, but do not prove, that GABAergic signaling through β3 containing GABA_A_-Rs in nonneuronal tissues is critical for normal development of the palate. An alternative explanation is that the time course and/or the extent of the genetic modification in the present study or in the study of Hagiwara et. al [28] was not sufficient to modulate palate formation. Thus, an obligate neuronal requirement of β3 for normal palate formation cannot be excluded. Continued investigation of β3's role in palate formation is warranted as the β3 locus has been implicated in human clefting defects [29].

The results presented here also shed new light on the requirement of β3 for normal viability. Global deletion of β3 resulted in a very high frequency of neonatal mortality, a result that is consistent with earlier studies of global β3 knockouts [18,27]. These results confirm that β3 is not required for survival *in utero *but is required for the transition to life immediately after birth. A high level (~61%) of perinatal mortality in pan-neuronal β3 knockouts was observed. The fact that pan-neuronal knockout did not result in cleft palate indicates that the perinatal mortality is not due to a defect in the palate that prevents feeding. The cause of death in these animals has not been determined. Furthermore, forebrain selective deletion of β3 (initiated ~2 weeks after birth) rescues the mice from perinatal mortality, but it fails to ensure survival to adulthood. Approximately 30% of forebrain selective β3 knockout mice die between 2 and 4 weeks of age. These results reveal that neuronal β3 is required beyond the perinatal period for normal viability. Furthermore, these results confirm that the mortality is not due to aberrant embryonic development. Instead, we conclude that there is a role for β3 in neuronal function that is required for survival.

Those conditional β3 knockouts that survived to adulthood displayed a variety of abnormalities that varied considerably between animals. Forebrain selective deletion of β3 also resulted in deficits in reproductive fitness, maternal care, and overt behavior. These phenotypes were similar to that observed in global β3 knockouts [3,14,27].

Hyperactivity is a common observation in global β3 knockout mice [3,18]. Although forebrain selective β3 knockout mice were not observed to be nearly as hyperactive as global knockouts, they exhibited a statistically significant increase in their total activity, compared to control mice (Figure 6A). As previous studies on global β3 knockout mice found them to exhibit high baseline velocities and ambulation [3], these experiments were designed to likewise separate out the ambulatory component of the total activity measurements on the conditional knockouts. Subsequently, there was a trend for ambulatory activity to be higher in forebrain selective β3 knockouts compared to cre- controls. This observation is evidence that locomotor components other than ambulation strongly impacted the resultant total activity measurements taken on the forebrain selective knockout mice. For example, the conditional knockouts, compared to controls, may have focused more on locomotor activities related to rearing, grooming or exploring small regions of the test apparatus rather than participating in higher ambulatory activity.

Body weights were increased by forebrain selective knockout of the β3 subunit in both male and female mice (Figure 5). This may have been due in part, at least in the males, to an increase in food intake and occurred despite an increase in locomotor activity. It is of interest to note that the response in both males and females was variable, with only a subset of animals showing a clear increase in weight. This is most likely due to the fact that animals in this study were of a mixed background. Given the large body of literature showing GABA or GABA_A_-R agonists to be stimulators of food intake [30], the increase in body weight was somewhat unexpected. It may be that deletion of the β3 subunit resulted in a compensatory GABA_A_-R response that increased the functional activity of GABA_A_-Rs in brain areas involved in regulating food intake, e.g., the hypothalmus. However, the role of GABA in the mechanisms governing food intake and body weight is unclear. GABA has been shown to be colocalized with neuropeptide-Y neurons in the arcuate nucleus of the hypothalamus [31] which contact and inhibit arcuate proopiomelanocortin neurons [32] that suppress food intake. We have observed that β3 is reduced ~50% in the hypothalamus of forebrain selective knockouts (data not shown). However, GABA_A_-R-containing neurons in the lateral parabrachial nucleus of the brainstem have also been postulated to be involved in GABA-mediated increases in food intake [30]. While GABA is clearly involved in regulating body weight, the ubiquitous nature of GABA in the brain makes determining the mechanisms involved difficult. The ability to manipulate expression of a predominant GABA_A_-R subunit in an anatomically-specific manner is an important first step in unraveling the role of GABA in regulating body weight.

A previous study revealed that global β3 knockout mice were less sensitive to the sedative/hypnotic effects of etomidate [33]. However, because of the ubiquitious nature of the β3 knockout, developmental compensation was observed [20] that made it impossible to determine if the reduction in anesthetic sensitivity was due to deletion of a direct target of etomidate or due to a compensatory change in an unknown target. In the current study, we demonstrated that inactivation of β3 selectively in forebrain neurons after ~2 weeks of postnatal life also resulted in reduced sensitivity to the sedative/hypnotic response to etomidate. This result, together with results derived from gene knockin studies [9,11] lead us to suggest that β3, and not a compensatory change in an unknown target, is a direct target for some of the anesthetic effects of etomidate.

Reportedly, β3 containing GABA_A_-Rs are also important for mediating the effects of ethanol [12]. However, the sedative/hypnotic effect of ethanol was not changed by conditional (Figure 3B, D) or global inactivation [33] of the β3 gene. Thus, the β3 subunit is not necessary for the sedative/hypnotic effect of ethanol. Other behavioral effects of ethanol (e.g., motor incoordination, cognative impairment, etc.) remain to be tested in these mice.

It is unknown why the cleft palate, mortality, body weight, and behavioral phenotypes are variably penetrant within the various β3 mutant mouse lines. It is possible that animal-to-animal variability exists because of genetic heterogeneity in the background of the animals. None of the mutant mouse lines used here were maintained on a uniform, inbred genetic background. Genetic background has been demonstrated to substantially alter the incidence of cleft palate in global β3 knockouts [28]. Detailed investigation of β3 mutants maintained on a hybrid genetic background could be used to search for modifier genes that interact with the GABAergic system in palate development (and possibly in the other observed phenotypes).

Alternatively, a possibility exists that those β3 mutant animals that escaped from palate defects and neonatal mortality were able to invoke a compensatory pathway to overcome the genetic defect. Indeed, global knockouts exhibited substantial compensatory responses [19,20]. Although deletion of β3 after birth would circumvent developmental compensation during early development, it is still possible (actually quite likely) that compensation also occurred in those mice. For example, we have observed that in the cortex of the forebrain selective knockouts, the amount of the α1 subunit of the GABA_A_-R was also reduced (Figure 4B). It is not clear if this is truly a compensatory change or if it reflects an obligate subunit partnership of β3 with α1 in this brain region. Nonetheless, while conditional knockout mice may circumvent some issues observed in global knockouts such as compensation during embryonic development, the conditional knockout approach is not without limitations.

The new line of genetically engineered mice described in this report, i.e., mice with a floxed β3 gene, will be invaluable for a wide variety of studies. As demonstrated in the current study, knockout of β3 by the traditional method of crossing the floxed mice to a cre recombinase expressing transgenic mouse can be used to make tissue specific and/or temporally regulated conditional knockouts. Addition of a regulated cre recombinase, such as cre-ER that is regulated by tamoxifen [34], would provide even greater temporal control over the conditional nature of the knockout. The floxed β3 mice will also be useful for nontraditional methods of creating conditional knockouts such as stereotaxic microinjection of cre expressing viral vectors [35] or in vivo electroporation of cre expressing plasmids [36]. This wide variety of approaches for producing mice that conditionally lack β3 will open up new avenues for investigating the role of β3 in normal physiology and pathophysiology.

## Conclusion

A mutant mouse line that harbors a floxed β3 subunit of the GABA_A_-R was successfully produced. Pan-neuronal and forebrain selective knockout of β3 revealed new insight into the role of the β3 subunit in palate formation, embryonic and postnatal viability, behavior, body weight regulation, and etomidate sensitivity. The floxed β3 mice described in this report will be very useful for conditional knockout studies to further investigate the role of the β3 subunit of the GABA_A_-R in various developmental processes, normal physiology, and pathophysiologic disorders such as Angelman syndrome and autism spectrum disorder.

## Methods

### Gene Targeting

A gene targeting DNA construct was prepared from Strain 129/SvJ mouse genomic DNA. This construct contained ~9.7 kb of mouse genomic DNA from the β3 locus, a loxP site inserted into intron 2, a neomycin selectable marker gene from pK-11 [22] that was flanked by two frt sites and a single loxP site (all inserted into intron 3), and the MC1-thymidine kinase gene [37]. The 5' loxP site was introduced into a novel BglII restriction site that was added to intron 2 by site directed mutagenesis (MORPH Mutagenesis Kit, 5 Prime-3 Prime, Inc., Boulder, CO). The neomycin cassette replaced a 321 bp BamHI fragment in intron 3. This construct was linearized with NotI and electroporated into R1 [38] embryonic stem cells as previously described [39]. Cells that survived G418 (265 μg/ml; Invitrogen) and ganciclovir (2 μM) selection were analyzed for gene targeting by Southern blot analysis. Correctly targeted clones were injected into C57BL/6J blastocysts to produce chimeric mice. Chimeras were mated to C57BL/6J mice to produce F1 animals that harbored the modified β3 locus. All animal studies followed the Guide for the Care and Use of Laboratory Animals and were approved by Institutional Animal Care and Use Committee's.

### Production of β3 Global Knockout Mice

Mice harboring the targeted, neo-containing floxed β3 locus were crossed to an actin-cre deleter mouse line (FVB X C57BL/6J background) [22] to produce a cre recombined β3 allele in all cells. Mice heterozygous for the cre recombined allele were interbred to produce wild type, heterozygous, and homzogyous global knockout mice.

### Production of β3 Conditional Knockout Mice

Mice harboring the neo-containing floxed β3 locus were crossed to an actin-FLPe deleter mouse line [21] to permanently and specifically delete the neo selectable marker from the β3 locus. The actin-FLPe mice originated on a B6SJLF2 genetic background but were backcrossed to C57BL/6J for 3 generations. Following FLPe-mediated deletion of the neo cassette from the β3 locus, the actin-FLPe transgene was bred out of the pedigree by backcrossing to C57BL/6J for one additional generation. Mice that were heterozygous for the floxed β3 locus without the neo gene were subsequently mated to Syn-cre [23,24] or α CamKII-cre (line T29-1) [25] transgenic mice to produce conditional β3 knockouts. Syn-cre mice were a gift of J. Marth (UCSD) and were received on a C57BL/6NHsd background that was backcrossed one generation to C57BL/6J. αCamKII-cre mice originated in the lab of S. Tonegawa (MIT) and were maintained in our lab on a C57BL/6J × Strain 129Sv/SvJ background.

### Western blot analysis

Protein levels in various brain regions of pan-neuronal and forebrain selective knockouts were analyzed using semi-quantitative Western blotting as described [40]. Briefly, cerebellum, cortex, and hippocampus were dissected and flash-frozen on dry ice. P2 membrane fractions were isolated and 25 ug of each sample was analyzed. Each sample (n = 4–5 samples/genotype) was analyzed on at least 2 different blots. Blots were sequentially probed with anti-β3 (NB300-119; Novus Biologicals, Littleton, CO) and anti-β-actin polyclonal (ab8227-50; Abcam, Cambridge, MA) antibodies. The actin signal was used to normalize for loading differences. Blots of cortex from forebrain selective knockouts and controls were also analyzed for GABA_A_-R α1 using an α1 specific antibody (Upstate, Lake Placid, NY). Primary antibodies were detected with HRP-conjugated goat anti-rabbit antibody (Novus Biolgicals) and visualized by chemiluminesence (Western Lightning, PerkinElmer Life and Analytical Sciences, Boston, MA). Immunoreactive protein levels were compared by densitometric measurement of band intensities and analyzed using Student's t test.

### Body weight and food consumption

Mice were individually weighed at weekly intervals between 4 and 16 weeks of age. Data were analyzed by repeated measures ANOVA.

Food consumption was determined on singly-housed 20 week-old male mice by weighing the amount of food consumed during an eight day period. The average daily food consumption (in grams) was calculated and data were analyzed using a Student's t test.

### Behavioral analysis

Adult forebrain selective and pan-neuronal knockout and control mice were tested for sensitivity to ethanol or the anesthetic etomidate using a standard sleep time assay as described [39]. Briefly, mice were injected with 3.5 g ethanol (Pharmco, Brookfield, CT) or 20 mg etomidate (Bedford Laboratories, Bedford, OH) per kg body weight into the intraperitoneal cavity. When mice lost the righting reflex, they were placed in v-shaped troughs and the time until they were able to right themselves three times within 30 sec was recorded. Sleep time was defined as the duration of the loss of the righting reflex. Differences between genotypes were determined using a t-test. Because of the limited number of mice available for this study, both males and females were used.

Operant learning was assessed using a step-through passive avoidance task as previously described in detail [41]. Briefly, a TruScan activity monitor (Coulbourn Instruments, Allentown, PA) that included a removable Plexiglas insert was used. The insert divided the test arena into light and dark chambers. The floor of the test apparatus was made of steel rods that were used to deliver a footshock. Mice were placed in the lighted chamber and the time to enter the dark side was recorded. Upon entering the dark chamber, the mice were delivered a mild footshock (0.4 mA, 3 sec), removed from the test apparatus, and returned to their home cage. Twenty-four hours later, the procedure was repeated except that no shock was delivered. The latency to enter the dark chamber was calculated as the difference in latency between day 2 and day 1. Separate groups of mice were treated exactly as described except they did not receive a footshock upon entering the dark chamber. These mice were referred to as the "no shock" group. Data were analyzed by ANOVA.

Spontaneous locomotor activity was assessed using an automated open field test apparatus. Mice were allowed to acclimate to the test room for 30 min prior to testing. Mice were placed individually into clear plastic monitoring chambers measuring 72 × 32 × 32 cm. Locomotor activity was measured via seven sets of photoelectric sensors evenly spaced along the length of the monitoring chamber, 4 cm above the floor of the chamber (San Diego Instruments, San Diego, CA). Total activity was recorded in arbitrary units reflective of the number of times a mouse interrupted the photoelectric sensors during a 10-min monitoring session. Data was automatically recorded and stored by computer. Data was collected in two bins, one representing the total activity and the other representing the ambulatory component of total activity. Total activity includes ambulatory activity as well as locomotor activities directed towards rearing, grooming and exploring small regions of the testing chamber. Data were analyzed by an unpaired Student's *t*-test using the software program PRISM 4 (GraphPad Software, San Diego, CA).

## Competing interests

The author(s) declares that there are no competing interests.

## Authors' contributions

CF and SLH performed experiments and analyzed data; DFW performed some western blot experiments and analyzed data; SMH directed body weight and food consumption studies; TMD performed and analyzed locomotor behavioral experiments; GEH conceived of the study, performed and/or directed experiments, analyzed data, and wrote the manuscript. All authors helped draft the manuscript and have read and approved the final version.
